# The human *O*-GlcNAcome database and meta-analysis

**DOI:** 10.1038/s41597-021-00810-4

**Published:** 2021-01-21

**Authors:** Eugenia Wulff-Fuentes, Rex R. Berendt, Logan Massman, Laura Danner, Florian Malard, Jeet Vora, Robel Kahsay, Stephanie Olivier-Van Stichelen

**Affiliations:** 1 Department of Biochemistry, Medical College of Wisconsin, Milwaukee, USA; 2 Department of Biochemistry & Molecular Medicine, The George Washington School of Medicine and Health Sciences, Washington, DC 20052 USA

**Keywords:** Glycoproteins, Glycosylation, Glycobiology, Nutrient signalling

## Abstract

Over the past 35 years, ~1700 articles have characterized protein *O*-GlcNAcylation. Found in almost all living organisms, this post-translational modification of serine and threonine residues is highly conserved and key to biological processes. With half of the primary research articles using human models, the *O*-GlcNAcome recently reached a milestone of 5000 human proteins identified. Herein, we provide an extensive inventory of human *O*-GlcNAcylated proteins, their *O*-GlcNAc sites, identification methods, and corresponding references (www.oglcnac.mcw.edu). In the absence of a comprehensive online resource for *O*-GlcNAcylated proteins, this list serves as the only database of *O*-GlcNAcylated proteins. Based on the thorough analysis of the amino acid sequence surrounding 7002 *O*-GlcNAc sites, we progress toward a more robust semi-consensus sequence for *O*-GlcNAcylation. Moreover, we offer a comprehensive meta-analysis of human *O*-GlcNAcylated proteins for protein domains, cellular and tissue distribution, and pathways in health and diseases, reinforcing that *O*-GlcNAcylation is a master regulator of cell signaling, equal to the widely studied phosphorylation.

## Introduction

The current trend of sugar-rich food intake poses a significant concern for public health worldwide. In the United States, the average US citizen consumes 17 teaspoons of added sugar every day^1^, whereas the American Heart Association (AHA) recommended upper limit is at most half of that. Increased sugar consumption correlates with increased diet-related diseases like obesity, diabetes, cardiovascular diseases, and other metabolic syndromes^2^. Therefore, understanding the molecular mechanisms through which the excess intake of sugar impacts metabolic regulation is critical to prevent and treat these diseases. Amongst the affected mechanisms is the modification of proteins by *O*-GlcNAcylation^3^.

**Fig. 1:**
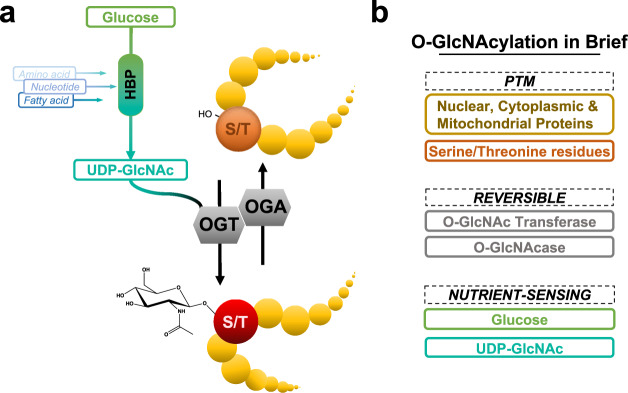
The *O*-GlcNAc cycling. (**a**) The *O*-GlcNAc cycling in humans. (**b**) An *O*-GlcNAc in Brief portion highlights the features and actors of *O*-GlcNAcylation. *HBP: Hexosamine Biosynthesis Pathway; OGT: O-GlcNAc Transferase; OGA: O-GlcNAcase; PTM: Post-Translational Modification; S/T: Serine/Threonine*.

The *O*-GlcNAc modification is a nutrient rheostat that transiently regulates functions, localization, and stability of proteins in response to fluctuations in nutrient intake (Fig. 1a,b)^4^. Indeed, the nucleotide sugar donor for this modification, UDP-GlcNAc, is the final product of the Hexosamine Biosynthetic Pathway (HBP). This pathway integrates carbohydrate, amino acid, nucleotide, and fatty acid metabolisms to maintain a suitable pool of UDP-GlcNAc. UDP-GlcNAc is then used for glycan synthesis, including *O*-GlcNAcylation catalyzed by the *O*-GlcNAc Transferase (OGT). On the other hand, the *O*-GlcNAcase (OGA) dynamically hydrolyzes *O*-GlcNAc, releasing the protein’s modification^4^.

Since its discovery in 1984, *O*-GlcNAc research has been thriving. On average, one article every two days has been published over the past decade (Figure S1a)^5^. However, the absence of an *O*-GlcNAc database has been narrowing our view of the field. Therefore, we combined the results from all *O*-GlcNAc articles published to date and objectively provide a comprehensive picture of this modification. We have created an extensive database of 5072 human *O*-GlcNAcylated proteins (available at www.oglcnac.mcw.edu) and performed a meta-analysis of surrounding amino acid sequences, protein domains, and molecular functions of these proteins in health and diseases.

## Results

### Over 5000 proteins and 7000 sites compose the human *O*-GlcNAcome

**Fig. 2:**
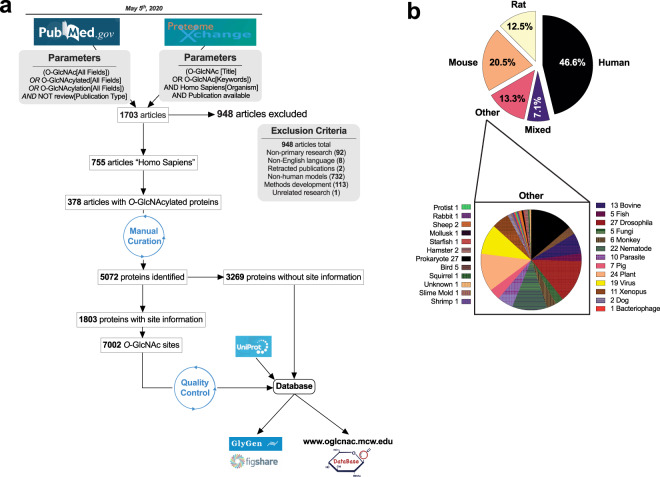
Literature search for *O*-GlcNAcylated proteins identified over 5000 human *O*-GlcNAcylated proteins. (**a**) Method flowchart and exclusion criteria. (**b**) Study models found in the *O*-GlcNAc literature.

About 1703 articles were extracted from a combined search on PubMed and Proteome Exchange (Fig. 2a, Figshare file 1^6^). A total of 948 articles were excluded from our list using the following criteria: non-primary research articles, non-English languages, retracted publications, and articles studying non-human proteins. About 85% of *O*-GlcNAc articles were either human-, mouse- or rat-based studies (Fig. 2b). Moreover, we observed *O*-GlcNAcylation in all seven biological kingdoms, highlighting the ubiquitous and conserved nature of this modification (Fig. 2b).

Of the 755 remaining articles, half of them identified human *O*-GlcNAcylated proteins (Fig. 2a, Figshare file 1^6^). Each *O*-GlcNAcylated protein was cataloged using their UniProtKB entry number as the primary identifier. References, techniques used, and *O*-GlcNAcylation sites were also documented. The list was further manually curated for duplicate entries and merged if identical. When possible, unreviewed UniProtKB entries were merged or replaced with reviewed entries. In the end, 5072 *O*-GlcNAcylated proteins were inventoried from the *O*-GlcNAc literature (Figshare file 2^6^). A subset of 1803 proteins contained 7002 distinct *O*-GlcNAcylation sites were identified through varied techniques. Therefore, this inventory is the most extensive library of *O*-GlcNAcylated proteins and site information made to date^7^. This list was first deposited to Figshare (#12443495) and GlyGen (#GLY_000517). In addition, we created an online platform to search the database in a more direct manner (www.oglcnac.mcw.edu).

For each entry, an *O*-GlcNAc score between 0 and 100, was attributed to each *O*-GlcNAcylated protein, reflecting the exhaustiveness of the *O*-GlcNAcylation description for each protein. The score combined the following parameters: (1) the timespan between the first and the last identification. Having a protein identified 20 years ago and still confirmed in recent analysis is a strong indication of reliability for the *O*-GlcNAc status; (2) the total number of references showing *O*-GlcNAcylation for a given protein; (3) the number of independent investigators (first and last authors) that reported the protein as *O*-GlcNAcylated, which limits the probability for errors; (4) the number of yearly citations for each paper was also added as an estimation of peers-validation of the publication in the field; (5) a modifier that underweighted papers with lots of proteins identified but few citation and overweighted publications that carefully characterized one protein and was well cited. By providing this *O*-GlcNAc score, we propose an unbiased meta-analysis to score the confidence in the identification of the *O*-GlcNAcylation on a protein. In our list, proteins with low score (<10) needs to be considered with caution and verified for their *O*-GlcNAcylation status.

From our inventory, most of the *O*-GlcNAcylated proteins were identified by MS-based studies, almost always combined with labeling strategies such as click-chemistry (4032/5072 proteins) (Figshare file 2^6^). This confirmed that the majority of the *O*-GlcNAcome has been discovered by mass spectrometry (Figure S1b). Indeed, due to technical limitations, *O*-GlcNAcylation was overlooked for a long time. However, reliable mass-spectrometry protocols lead to proper and efficient mapping of cellular *O*-GlcNAcylation. Besides, the sub-stoichiometric nature of the modification still necessitates some enrichment before identification, such as lectin or enzymatic labeling and pull down. As a result, of the 1803 proteins with site information, only 15 used non-MS strategies, often opting for *in silico* analysis, pull-down and mutagenesis (Figshare file 2^6^).

### Analysis of human O-GlcNAc sites refined the *O*-GlcNAcylation semi-consensus sequence

Over the past decades, 7002 *O*-GlcNAcylation sites were characterized in 1803 proteins (Fig. 2a, Figshare file 2^6^). Of those, 58.4% were found on serine as opposed to 41.6% threonine residues. However, this follows the relative abundance of serine/threonine in the human proteome (S:60.9%/T:39.1%) (https://www.uniprot.org/uniprot/?query=proteome:UP000005640). It suggests that the OGT has little to no preferences toward one residue over the other.

Heavily modified proteins (at least 50 sites) were identified as (1) involved in the formation of stress granules, including UBP2L (88 sites) and PRC2C (68 sites); (2) part of the pre-synaptic interface, including Bassoon (73 sites) and PCLO (62 sites); and (3) part of the nuclear transport complexes, including Host Cell Factor 1 (HCF1) (169 sites), Nucleoporin 214 (Nup214) (177 sites), Nup153 (131 sites), Nup98 (91 sites), Nup62 (87 sites) and Nup58 (53 sites) (Figshare file 2^6^). So far, the latter are the most *O*-GlcNAcylated proteins described to date.

**Fig. 3:**
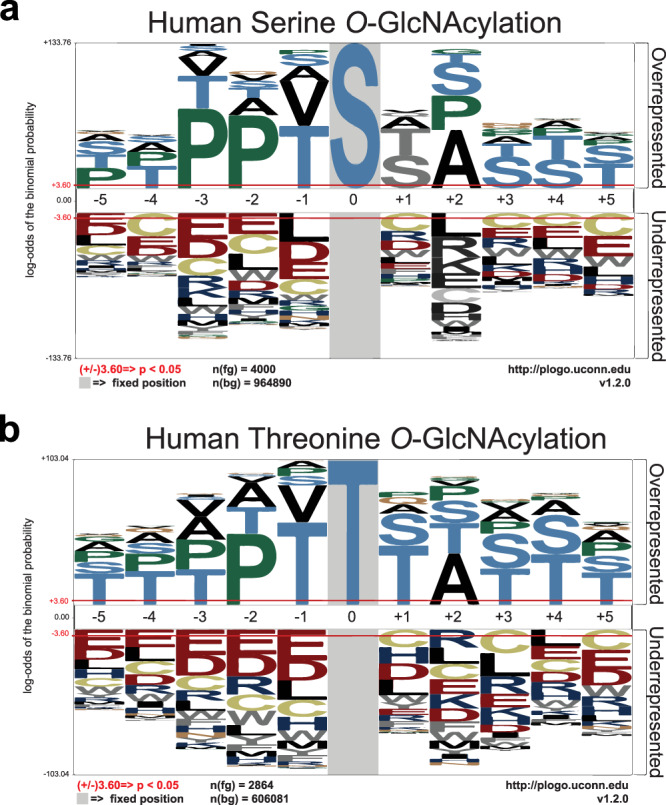
*O*-GlcNAcylation semi-consensus sequence. (**a**) Serine and (**b**) Threonine *O*-GlcNAcylation semi-consensus sequences based on the 7002 human *O*-GlcNAcylation sites surrounding sequences.

*O*-GlcNAc is a signaling regulator more akin to phosphorylation, despite some distinct characteristics. Whereas protein phosphorylation is governed by about 518 specific kinases and about 200 phosphatases^8,9^, only two enzymes regulate the addition and removal of *O*-GlcNAc from thousands of protein targets. Therefore, while phosphorylation sites can be well predicted based on consensus sequences, *O*-GlcNAc sites do not have a strict consensus. To refine the preferred sequence for *O*-GlcNAcylation, we performed a sequence alignment on the 7002 *O*-GlcNAc sites in this study (Fig. 3/Figshare file 2^6^). An abundance of serine or threonine residues around the *O*-GlcNAc sites was overall advantageous. On the contrary, the presence of glutamic acid or cysteine was mostly unfavorable to *O*-GlcNAcylation. The following sequences were extracted as the semi-consensus sequence for both serine and threonine *O*-GlcNAc modification in human proteins (Fig. 3):

$${\bf{P}} \mbox{-} {\bf{P}} \mbox{-} ({\bf{V}}/{\bf{T}}) \mbox{-} {\bf{g}}({\bf{S}}) \mbox{-} ({\bf{S}}/{\bf{T}}) \mbox{-} {\bf{A}}.\quad \quad ({\bf{P}}/{\bf{T}}) \mbox{-} {\bf{P}} \mbox{-} ({\bf{V}}/{\bf{T}}) \mbox{-} {\bf{g}}({\bf{T}}) \mbox{-} ({\bf{S}}/{\bf{T}}) \mbox{-} ({\bf{A}}/{\bf{T}})$$

### Cellular distribution analysis confirmed *O*-GlcNAcylated proteins are concentrated in nuclear and cytoplasmic compartments

**Fig. 4:**
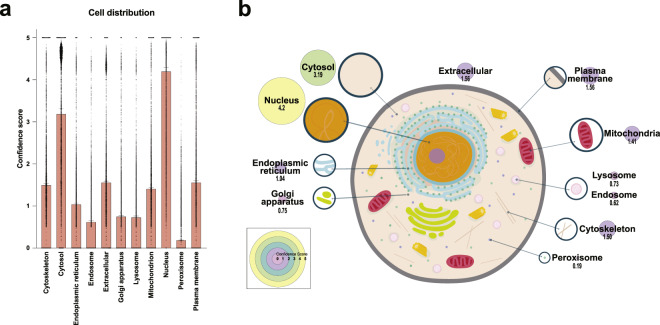
Cellular distribution of the *O*-GlcNAcome proteins. (**a,b**) Cellular localization of the human *O*-GlcNAcylated proteins (n = 4969). (**a**) The median and 95% Confidence intervals are represented. (**b**) Circle sizes and colors represent the median confidence score.

The 5072 *O*-GlcNAcylated proteins were submitted to the STRING database, and cellular distribution information was extracted, including a confidence score for each protein and compartment ranging from 0 (not present) to 5 (highly significant)^10^. As previously highlighted in the literature, *O*-GlcNAcylated proteins were mainly nuclear and cytoplasmic, with a median confidence score of 4.20 and 3.19, respectively (Fig. 4, Figshare file 3a^6^). Although some mitochondrial proteins were *O*-GlcNAcylated^11^, the median confidence score for that compartment was only 1.41. Therefore, only a small percentage of human *O*-GlcNAcylated proteins are mitochondrial compared to the nucleus or cytoplasm.

### Tissue distribution underlined the strong characterization of brain and liver O-GlcNAcylation

**Fig. 5:**
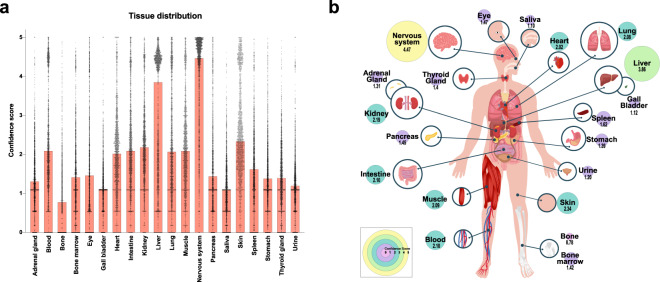
Tissues distribution of the human *O*-GlcNAcome (**a,b**) Tissue distribution of the human *O*-GlcNAcylated proteins (n = 2052). (**a**) The median and 95% Confidence intervals are represented. (**b**) Circle sizes and colors represent the median confidence score.

To understand where *O*-GlcNAc deregulation would have the most impact, we studied the organ distribution of *O*-GlcNAcylated proteins^12^. Like cell compartment distribution, a confidence score was attributed for each *O*-GlcNAcylated protein, and the median was represented for each organ (Fig. 5/Fighare file 3b^6^). *O*-GlcNAcylation was found in all major organs. However, tissue distribution interestingly correlated with high interest organs in the field as well as the availability of those tissues to a vast number of researchers. Therefore, the most *O*-GlcNAcylated proteins were identified in the brain and liver, with respective scores of 4.47 and 3.86. This distribution correlated with the tissue distribution of OGT^13,14^. Most organs showed some level *O*-GlcNAcylated proteins. The least *O*-GlcNAc abundant sites in the human body were bones (0.78), saliva (1.10), gall bladder (1.12), and urine (1.20). In addition to being rarer and harder to process, those sites are also composed of generally fewer cells, which therefore would be overall limited in intracellular proteins. This analysis emphasized some understudied tissues that could be of high significance such as the pancreas. As new studies emerge, this distribution will be refined through the web interface.

### Enrichment analyses highlighted the role of *O*-GlcNAcylation in regulating genetic information and cell signaling

**Fig. 6:**
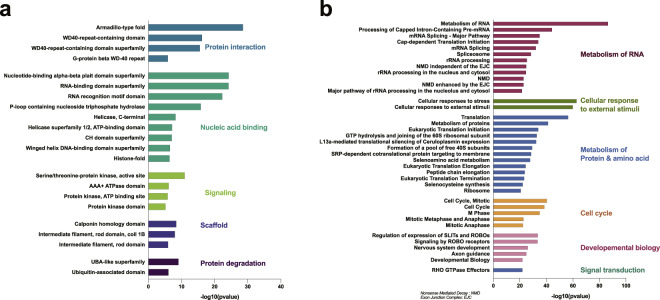
Protein domains and pathways enrichment of the human *O*-GlcNAcome. (**a**) Protein domain enrichment analysis of the human *O*-GlcNAcome. (**b**) Pathway enrichment analysis of the human *O*-GlcNAcome (n = 5059).

We then performed an enrichment analysis for protein domains (InterPro) and signaling pathways (KEGG and Reactome) on the human *O*-GlcNAcome (Fig. 6/Figshare file 4a/b^6^). For both protein domains and pathways, the enrichments highlighted the importance of *O*-GlcNAcylation in controlling genetic information and regulating protein signaling.

1. *Protein interaction and cell signaling:* Protein domains and pathway enrichment emphasized a central role for *O*-GlcNAcylation in signaling. Indeed, the most enriched domains types in human *O*-GlcNAcylated proteins were “Protein Interaction” and “Signaling” and included domains such as the Armadillo, WD40 (propeller-like arrangements of 4–8 β-folds) and kinase domains (Fig. 6a/Figshare file 3a^6^). Since these domains present great interaction surfaces, proteins with these domains are usually vital for signal transduction. Therefore, *O*-GlcNAcylated proteins in this category were central to signaling pathways and included β-catenin, the regulatory-associated protein of mTOR (RPTOR), Receptor of activated protein C kinase 1 (RACK1), and the histone-binding protein RBBP4 (Figshare file 2^6^). In this category, essential protein transporters like importins were also enriched. In the pathway analysis, cellular response to stimuli or stress, cell cycle, and development were significantly enriched in *O*-GlcNAcylated proteins (Fig. 6b/Figshare file 4b^6^). Indeed, *O*-GlcNAc-modified proteins were involved in cellular responses to various external stimuli, including hypoxia and heat stress or response to amino acid deficiency^15^. Heat-shock proteins (HSPs) constituted part of this category. Finally, many actors of cell cycle and development were also enriched, including Cyclin D, Retinoblastoma protein (RB), Minichromosome Maintenance proteins (MCM3,6,7), Vimentin, Enhancer of Zeste 2 Polycomb Repressive Complex 2 Subunit (EZH2), and Transcriptional repressor protein Yin and yang 1 (YY1) (Figshare file 2^6^).
2. *Nucleic acid-binding and metabolism of RNA:* Both protein domain and pathway enrichments emphasized the predominant role of *O*-GlcNAcylation in the control of genetic materials. *O*-GlcNAcylated proteins often presented nucleic acid-binding domains, including DNA-binding (histone-fold, TATA-box binding protein (TBP) domain) and RNA-binding (nucleotide-binding α/β plait superfamily, RNA recognition motif domain, K homology domain, ribosome, translation protein beta-barrel domain, and the ribosomal protein S5 domain 2-type) (Fig. 6a/Figshare file 4a^6^). Therefore, a large portion of the human *O*-GlcNAcome contributed to RNA metabolism, as demonstrated by pathway enrichment analysis (Fig. 6b/Figshare file 4b^6^). This included mRNA processing and splicing, nonsense-mediated decay (NMD), and ribosomal RNA (rRNA) processing. Amongst the *O*-GlcNAcylated proteins identified in those categories were the transcriptional repressor EWS, Enhancer of mRNA-decapping protein 3 (EDC3), GTP-binding nuclear protein Ran, Heterogeneous nuclear ribonucleoproteins (hnRNPs), Nuclear RNA export factor 1 (NXF1), Histones (2 A/2B/3/4), TBP, and RNA polymerase II (Figshare file 2^6^).
3. *Metabolism of Protein:* The human *O*-GlcNAcome was enriched for proteins participating in the processing or degradation of proteins, including ribosomal and proteasomal subunits (Fig. 6a/Figshare file 4a^6^). Pathway enrichment analysis also emphasized various aspects of protein metabolism, including the translational machinery (initiation, elongation, termination), co-translational protein targeting, and translational silencing of stress response gene expression (Fig. 6b/Figshare file 4b^6^). Examples in this category were regulatory subunits of the proteasome (non-ATPase, 26S and 11S), 40S ribosomal proteins S2 and S16, RACK1, translation initiator eIF4 A, F, G, and translational co-factor p67 (Figshare file 2^6^).
4. *Cellular structure:* Scaffold protein domains, including the calponin homology domain and actin-binding domain, were also found enriched in the human *O*-GlcNAcome. This group contained *O-*GlcNAcylated plastinins and actinins (Figshare file 2^6^), which correlated with the partial localization of *O*-GlcNAcylated proteins in the cytoskeletal fraction (Fig. 4/Figshare file 3a^6^), pointing toward a more structural role for *O*-GlcNAcylation.
5. *Disordered domains*: Using the DisProt database, we also observed that *O*-GlcNAcylation was prevalent in intrinsically disordered proteins (Figshare file 4c^6^)^16^. Out of the 591 manually curated human proteins available in the database, 50% were *O*-GlcNAcylated proteins, including the isoform Tau-F, α-synuclein, and CDK1 (Figshare file 4c^6^). The average disorder content of those *O*-GlcNAc proteins was 26%.

## Discussion

This article generated an extensive inventory serving as a standing database for human *O*-GlcNAc-modified proteins with 5000+ proteins and 7000+ *O*-GlcNAc sites available in the literature. In addition to being deposited to Figshare and GlyGen, we developed a web interface to interrogate the database (www.oglcnac.mcw.edu). Consequently, we encourage feedback and requests for integration of new proteins using the website’s submission tool. For each protein, an *O*-GlcNAc score describes the extent of the identification and is, therefore, an estimator of the confidence. There is no strict threshold to validate a protein’s *O*-GlcNAcylation. However, caution is advised for low score proteins (<10) for which extra-validation should be performed. Still, site mapping is the most decisive proof of *O*-GlcNAcylation in the *O*-GlcNAc field, which was only found in 35.5% of the human *O*-GlcNAcylated proteins.

Based on 7002 sites, the sequence analysis suggested that although there is no consensus sequence for *O*-GlcNAcylation, some amino acids are found more frequently. With more than twice the input sequences, our findings corroborate previously published *O*-GlcNAcylation semi-consensus sequences^17–21^. They can be used to refine existing or create new *in silico* prediction tools for *O*-GlcNAcylation.

Previously, gene ontology already suggested the enrichment of *O*-GlcNAcylated protein on RNA binding/processing proteins in activated human T cells and *Arabidopsis thaliana*^22,23^. Based on our extensive inventory of human *O*-GlcNAcylated proteins, we confirmed that a predominant role for *O*-GlcNAcylation lies in controlling genomic information, likely in response to nutrient input. This function is accomplished by the RNA/DNA-binding ability of many *O*-GlcNAcylated proteins and their RNA metabolism action. Predominantly, *O*-GlcNAcylation regulates the activity of numerous transcription factors, modifies the histones, and regulates RNA polymerase II activity^24–26^. Interestingly, *O*-GlcNAcylation also controls intron retention on various mRNAs, including *OGT* and *OGA* themselves, allowing the enzymes’ rapid production in response to nutrient input^27,28^. Consequently, acute OGT inhibition affects the spliceosome/RNA-splicing pathway, and particularly the ability to retain introns^28^.

Expected of post-translational modifications, *O*-GlcNAcylation is also involved in regulating protein production and processing, including the translational machinery itself. Both OGT and OGA are tightly associated with ribosomes. Overall, *O*-GlcNAcylation enhances translation complex formation and stability by modifying ribosomal proteins, co-factors such as RACK1^29^ or p67, and the translation initiation complex eIF2^30–32^. Finally, *O*-GlcNAcylation also prevents protein degradation by modifying subunits of the proteasome^33^. Altogether, *O*-GlcNAcylation is involved in every step of expressing our genetic information, from the activation or repression of genes, the production of mRNA and protein, and their processing, which overall defines cellular functions.

The responsiveness of *O*-GlcNAcylation to nutrient input is a critical characteristic of this modification. It allows for regulation of various cell signaling cascades in response to environmental changes. For example, the ability to thrive in favorable conditions engages *O*-GlcNAc cycling in many aspects. *O*-GlcNAcylation levels vary during the cell cycle, and its alteration disturbs mitotic phosphorylation, cyclin expression patterns, and cytokinesis, ultimately resulting in cell cycle failure and cell death^34^. Additionally, many cell cycle actors (cyclins, Cyclin-dependent Kinases) and pluripotency factors (OCT4 and SOX2) are modified in embryonic stem cells (ESCs), where *O*-GlcNAcylation regulates their transcriptional activity^35^. Finally, in Drosophila, *Ogt* is encoded by the essential Polycomb Group (PcG) gene *super sex comb* (*sxc*)^36^, crucial for proper patterns of development. *O*-GlcNAcylated proteins are also enriched on Polycomb response elements (PREs)^37^. OGT interacts with the Polycomb repressive complex 2 (PRC2) in human cells, one of the two PcG protein complexes in mammals^38^. The tight control of *OGT* expression during development by X-inactivation^39^ also emphasizes the importance of OGT regulation during development. Altogether, those studies collectively support the vital role that *O*-GlcNAc plays in regulating the cell cycle and embryonic development.

Furthermore, the ability to quickly respond to stress is also a feature in which *O*-GlcNAcylation is involved. A global increase of *O*-GlcNAc levels is shown in response to several models of cellular stress induction^40–42^. Those studies led to the identification of HSP-70 family members as *O*-GlcNAc-binding lectins^43^. *In vivo* studies in trauma-hemorrhage and ischemia/reperfusion injury models showed decreased *O*-GlcNAc levels following stress injury with subsequent necrosis and apoptosis^44,45^. Taken together, these data suggest that *O*-GlcNAcylation plays a protective role in response to cellular stress.

Across the years, the essential nature of *O*-GlcNAcylation has been observed in human and animal models. In humans, OGA or OGT deletions are not viable. Indeed, *OGT* is necessary for ESC viability, and conditional *Ogt* knockout is lethal in mice^46^. On the other hand, conditional deletion of *Oga* in mice is perinatal lethal, leading to severe phenotypes including altered glucose homeostasis, metabolic defects, and deregulation of genes related to growth and metabolism^47^. OGA inhibition leads to impaired differentiation of mESCs and activates transcription of genes usually epigenetically repressed in mESCs^48^. Recently, OGT deregulation has been identified in patients with X-linked Intellectual Disability (XLID) patients, accentuating once more the importance of *O*-GlcNAcylation in the brain. In those patients, OGT’s single nucleotide polymorphism generates improper *O*-GlcNAc and OGA levels^49–51^, but does not completely abolish *O*-GlcNAcylation.

It is now widely accepted that balanced levels of *O*-GlcNAcylation are necessary for normal cell physiological function and that deregulation of those levels leads to diseases. The brain is the organ concentrating the most *O*-GlcNAcylated proteins in humans so far and is also one of the most glucogenic organs. Not surprisingly, the brain consumes about 120 g of glucose daily^52^. In a resting state, up to 60% of the whole-body glucose is used by the brain. The central nervous system is home to many *O*-GlcNAcylated proteins, including Microtubule-associated protein Tau, α-Synuclein, β-Amyloid precursor protein (APP), and Huntingtin (Figshare file 2^6^). These proteins are linked to various neurodegenerative pathologies, including Alzheimer’s, Parkinson’s, and Huntington’s diseases. Alteration of *O*-GlcNAcylation levels has been extensively linked to neurodegeneration^53^. Increased *O*-GlcNAcylation is typically protective in patients with neurodegenerative diseases. The human Tau protein carries *O*-GlcNAc residues that compete with key phosphorylation residues and prevents its aggregation^54^.

Similarly, *O*-GlcNAc protects neurons from Parkinson’s disease by modifying α-Synuclein and preventing its aggregation. Finally, *O*-GlcNAcylation on APP decreases the production of β-Amyloid peptides and reduces the formation of amyloid plaques^55^. *O*-GlcNAcylation in neurodegenerative diseases is the hotspot of *O*-GlcNAc research with promising treatment options emerging^56,57^.

The liver is a central platform for glucose metabolism in the human body and, as a result, shows a high concentration of *O*-GlcNAcylated proteins. The physiological role of *O*-GlcNAcylation in glucose metabolism includes the modification and regulation of the Insulin receptor, IRS1, AKT, GSK3β, FOXO1, and PGC1α (Figshare file 2^6^). In mice, alteration of *O*-GlcNAc enzymes in the liver causes insulin resistance and dyslipidemia^58^, which is also the main feature of Non-Alcoholic Fatty Liver Disease (NAFLD). Increasing *O*-GlcNAcylation is protective against hepatic steatosis, supporting the critical role of *O*-GlcNAc in the liver^59^. Finally, liver fibrosis, one of the symptoms of Non-Alcoholic Steatohepatitis (NASH), is prominent in mice depleted of *O*-GlcNAcylation in the liver^60^. Similar to the brain, these studies suggest a protective role for *O*-GlcNAcylation in the liver.

In light of the significant advances in the field and the recent milestone of 5000 human proteins identified, *O*-GlcNAcylation is a highly prevalent area for biomedical research. Furthermore, it is even studied as a treatment option, for example, in neurodegenerative diseases^56,57,61,62^, increasing its significance for the medical community. With more technical innovations and growing interest in *O*-GlcNAc research, the role of *O*-GlcNAcylation in common pathologies is expected to be the focus of more intensive research in the near future.

## Methods

### Literature search

To identify *O-*GlcNAcylated proteins, a systematic literature search was conducted (Fig. 2/Figshare file 1^6^). PUBMED search was finalized on May 5^th^, 2020, with the following search terms: “*O-*GlcNAc,” “*O-*GlcNAcylated,” and “*O-*GlcNAcylation” to englobe the entire *O*-GlcNAc literature. Previous reviews were excluded by adding the terms “NOT review” in the search criteria (Fig. 2). No restriction was imposed on the publication date.

### Proteome exchange search

To identify more *O-*GlcNAcylated proteins, we have also included a search from Proteome Exchange (http://www.proteomexchange.org/), repository for mass spectrometry data. Our search criteria were “*O*-GlcNAc” in either the “Title” or “Keywords” field and “Homo Sapiens” in the “Species” field. Only datasets with a publication available were retained in our initial literature list.

### Literature curation

The search results were then compiled and screened manually by the authors. Papers were rejected if they met any part of the exclusion criteria (Fig. 2). In short, non-primary research, non-English language, and retracted articles were excluded. Similarly, a study in which the source of the protein used could not be identified was also excluded. Publications detailing novel techniques and methods or computational studies were excluded unless they showed direct protein *O-*GlcNAcylation. Finally, articles were excluded when no direct evidence of protein *O-*GlcNAcylation was shown.

### Human research curation

Only *O*-GlcNAcylated human proteins were retained for the final *O*-GlcNAcome list. The research was considered human if human proteins were used to study the *O*-GlcNAc status, whether endogenous, purified, or recombinant. Studies using non-human proteins in a human-based cell system were excluded and considered non-human models. Publications showing the modification of human origin proteins and non-human proteins were included and classified as “human mixed.”

### *O*-GlcNAcylated protein curation

A total of 5072 *O*-GlcNAcylated human proteins were extracted and cross-referenced, when available, to UniProtKB Entries. For each protein, the UniProt accession number (AC), Entry name, protein name, gene names, curation status (“Reviewed” *vs*. “unreviewed”), modification sites, and identification techniques used were documented. Duplicate entries were merged and updated with any new information discovered throughout curation. Identified proteins from experiments that included protein IDs were also cross-referenced with UniProtKB. The list was then manually screened a second time to further improve accuracy by increasing the number of curated UniProtKB entries. “Unreviewed” UniProtKB entries were replaced or merged with corresponding “reviewed” entries when available. When multiple UniProtKB entries were available, “reviewed” entries were preferred to “unreviewed.” “Unreviewed” entries sharing gene names with “reviewed” entries were merged into the appropriate “reviewed” UniProt KB entry. Finally, any proteins identified by articles as fragments, and therefore “unreviewed,” were replaced with the full-length protein entry when available. The final table constitutes the human *O*-GlcNAcome database (Figshare file 2^6^).

### *O*-GlcNAc sites quality control

A python script in the GlyGen^63,64^ backend pipeline was used to process each protein entry of the database table and perform quality control (QC). In the QC checks, the reported UniProtKB accessions in the database were matched with the GlyGen’s human protein master list of UniProtKB canonical accessions to ensure all of the proteins belong to the human species. Next, the *O*-GlcNAc site data that contained the amino acid residue and its position were mapped to the GlyGen’s FASTA sequence for the corresponding human UniProtKB canonical proteins. The amino acid residues and positions that did not match with the FASTA sequence of the canonical protein were flagged and excluded from the table. Similarly, protein entries that did not have any site information were also excluded. The entries that passed all the quality control steps were exported as a final CSV dataset file. In contrast, the excluded entries were exported in a log file with the reasons for exclusion for further manual verification.

When site information differed from the original publication to align the UniprotKB canonical sequence, a disclaimer was added, and the changes were indicated in the note section.

### *O*-GlcNAc Score

The *O*-GlcNAc score was assigned for each protein identified (Figshare file 2^6^). The score was calculated based on the following equation, in which all components were normalized by their maximal value in the dataset of 5072 entries:

$$S\left(x\right)=R{\left(x\right)}^{norm}+C{\left(x\right)}^{norm}+T{\left(x\right)}^{norm}+fA{\left(x\right)}^{norm}+lA{\left(x\right)}^{norm}+B{\left(x\right)}^{norm}$$

Briefly, given *x* the list of all protein entries and *x* a single entry, and considering the list of references *Nx* and *P* the number of protein entries documented in an index *i* of *Nx*:

- *R* is the length of the list of references *Nx*.
- *C* is the sum of per-year citations for each index *i* of *Nx*.
- *T* is the time span between the first and last reference publication.
- *fA* and *IA* are the number of distinct first and last author, respectively, within *Nx*.
- *B* is a bonus term computed for each index *i* of *Nx* and averaged over *R*. Higher *Pi* negatively impacts *B* whereas higher *Ci* positively impacts *Bi*.

This absolute score is theoretically contained in the [0,6] range. We converted in a relative scale by normalizing score values over the top score protein entry.

### *O*-GlcNAc semi-consensus sequences

*O*-GlcNAcylation semi-consensus sequence was generated after quality control using the −5 to + 5 amino-acid sequence surrounding the 7002 *O*-GlcNAc sites. A frequency graph was generated using pLogo^65^.

### String network mapping

Cytoscape was used to analyze the list of proteins using the STRING database^66^. Briefly, the UniProtKB ID list was imported using the “Import Network from public database” function and selecting the “STRING: protein query” option. *Homo sapiens* was chosen as the target species, and the following parameters were applied to create the STRING network: “confidence (score) cutoff”: 0.40; “Max additional interactors”: 0. A total of 4975 proteins matched to the STRING database and were used for the distribution and enrichment analysis. Due to the large number of proteins to analyze, four-node tables were generated and compiled together.

### Cellular and tissue distribution

From the final node table (Cytoscape), UniProtKB ID and confidence score for each “Compartment” and “Tissue” column were extracted (Figshare file 3a/b^6^). Empty cells were assigned a 0 score, the lowest confidence score. A score of 5 corresponded to the highest confidence^10,12^. A total of 2923 or 6 entries did not have tissues or compartment information, respectively, and were therefore excluded from the analysis. For each compartment or tissue, the median confidence score was used for the analysis and graphic representation.

### Enrichment analysis

To perform enrichment analysis of the human *O*-GlcNAcome, the HumanMine server^67^ was used. Similarly, 5059 protein/gene couples matched the various databases used to perform enrichment analysis (Figshare file 4a/b^6^). Protein domain enrichment was performed using the following parameters: Test correction: Holm-Bonferroni; Max p-value: 0.05; Background population: Default. Gene ontology enrichment was also performed for molecular function using similar parameters. Finally, pathway enrichment for KEGG and Reactome datasets was performed using identical parameters. For all analyses, p-values were extracted and plotted.

### Intrinsic disorder protein domain

The total 5072 human *O-*GlcNAcylated protein entries were mapped to the DisProt database^16^ (Figshare file 4c^6^). The human database contains 591 manually curated intrinsically disordered proteins, and 299 DisProt entries were mapped as *O*-GlcNAcylated.

### Website

A website interface was created to browse through the database and submit comments and request for integration (www.oglcnac.mw.edu).

It relies on the non-relational database management system MongoDB and is based on the Django web framework for rendering (https://www.djangoproject.com/). Backend processes were all developed using the Python programming language (v3.7) and the pymongo library (https://pypi.org/project/pymongo/) for database server-client interactions. GNU/Linux Debian-based systems with gunicorn (Python http) (https://gunicorn.org/) and NginX (SSL/reverse proxy) (https://www.nginx.com/resources/wiki/) were used for development and production of the O-GlcNAc Database.

## Supplementary information

Figure S1

## Data Availability

The database can be accessed through the web platform www.oglcnac.mw.edu. The *O*-GlcNAcome repository was also deposited on Figshare under the 10.6084/m9.figshare.12443495.v146. Finally, the full curated repository was also deposited and integrated into the GlyGen database under the URL: https://data.glygen.org/GLY_000517, under Creative Commons Attribution (CC BY 4.0) license. The URL is linked to the dataset’s entry page. It contains the download option to download the dataset, BioCompute Object, and README that provides the bioinformatics workflow and metadata details.

## Code availability

Source code for the GlyGen QC and integration can be found in the Github repository: https://github.com/glygener/glygen-backend-integration/blob/master/pipeline/integrator/make-proteoform-dataset.py
