# Supplementary material for: The human O-GlcNAcome database and meta-analysis
Source: Sci Data. 2021 Jan 21;8:25. doi: 10.1038/s41597-021-00810-4 (PMC7820439; doi:10.1038/s41597-021-00810-4)
Supplement: Supplementary file 1 — Figure S1 [file 41597_2021_810_MOESM1_ESM.pdf]

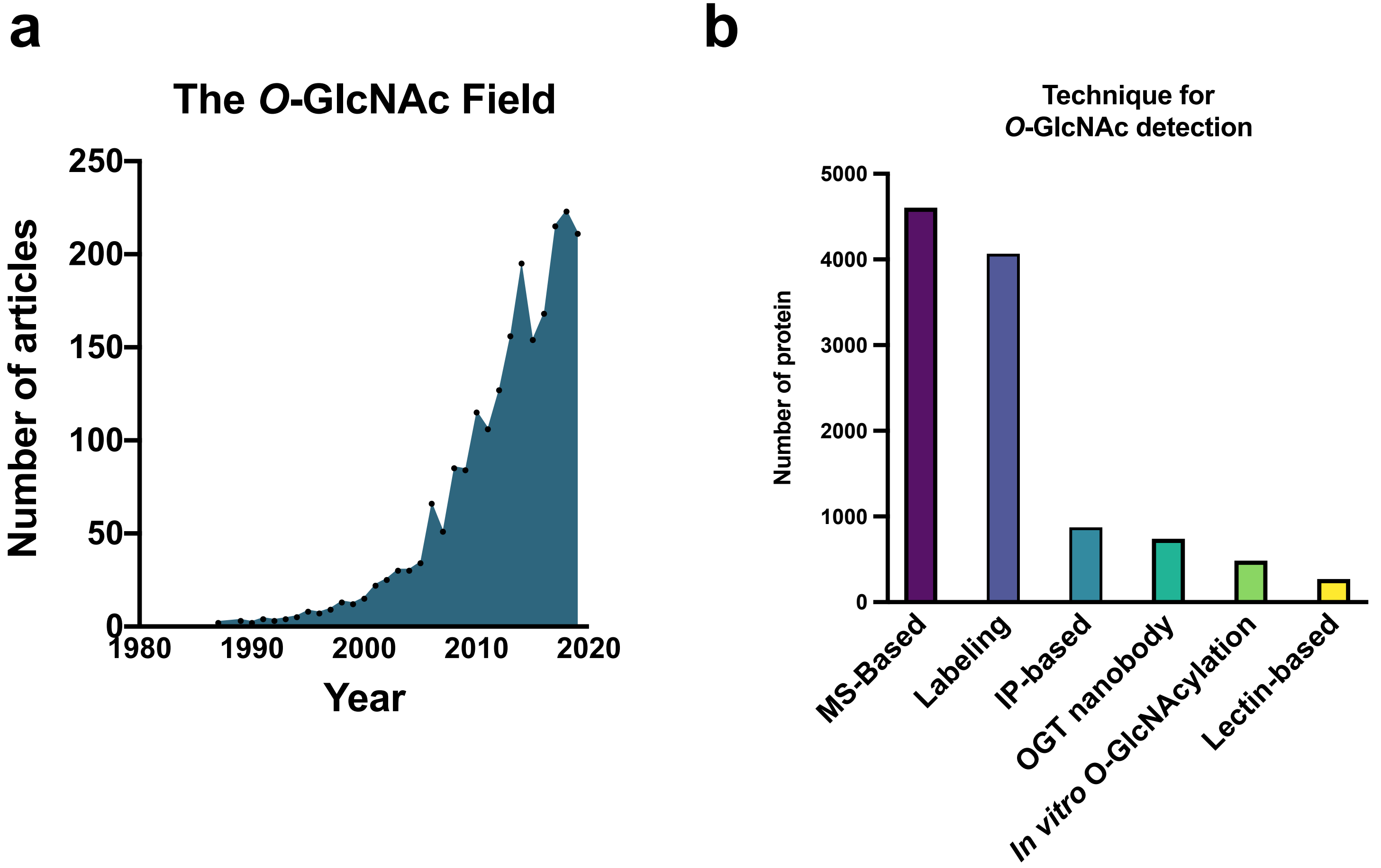

<u>Figure S1</u>: State of the *O*-GlcNAc field. a) Number of publications per year on O-GlcNAcylation. b) Methods used to identify human O-GlcNAcylated proteins.
